# Supplementary material for: Chromas from chromatin: sonification of the epigenome
Source: F1000Res. 2016 Mar 3;5:274. [Version 1] doi: 10.12688/f1000research.8001.1 (PMC4805159; doi:10.12688/f1000research.8001.1)
Supplement: Peer review history

# Peer review: Referee response for version 1

**DOI:** 10.5256/f1000research.8610.r12734

**Published:** 2016-03-22

**Authors:** Federico M Giorgi

**Affiliations:** 1 Department of Systems Biology, Columbia University, New York, NY, USA

The article "Chromas from chromatin: sonification of the epigenome" proposes a new and original method to sonify (i.e. convert into music) data information coming from the analysis of the epigenome.

The paper is extremely well written and it should be indexed on the basis of being the first to try this kind of review. The results of Cittaro *et al.*'s work, although without an immediate applicability in the field of biomedicine, is by itself a small scientifical breakthrough.

My only concerns regard the applicability of the sonification method as a truly alternative way to detect biological properties.

The authors imply that similar biological features (e.g. belonging to a particular pathway,  differential expression) could be discerned by listening to gene-centered audio tracks. I listened to such tracks and I concur, but to be scientifically complete, the authors may think (even as a follow up paper) to test this on a wider subject set. I would propose a psychological study with a blind panel of human subjects, who are then asked, after listening to some biological properties on a "training set", to find genes with similar properties on a "test set".

I have read this submission. I believe that I have an appropriate level of expertise to confirm that it is of an acceptable scientific standard.

# Peer review: Referee response for version 1

**DOI:** 10.5256/f1000research.8610.r12735

**Published:** 2016-03-18

**Authors:** Ho-Ryun Chung

**Affiliations:** 1 Otto-Warburg-Laboratory, Max Planck Institute for Molecular Genetics, Berlin, Germany

“Chromas from chromatin: sonification of the epigenome” by Cittaro *et al*. deals with the transformation of ChiP-seq profiles into music. The authors transform BigWig files into notes by discretizing the logarithm of the signal intensity. Test individuals perceive the resulting music as more appealing than randomized controls. The authors explore the possibility to use the music to discern differentially expressed genes in two cell lines from unchanged ones. The classification accuracy is a little bit better than random guessing. They also use the similarity of the music to cluster genes. They claim that the clustering reveals genes with similar biological properties. They also show that the clustering is correlated to the gene expression level.

I find the idea really interesting. It may help the vision impaired to gather information about ChIP-seq tracks or other quantitative vector like information. It may be used to train individuals to recognize certain epigenomic features, like promoters and enhancers.

I find it not so surprising that the chromatin music is more appealing to test persons than randomized controls, because of the smoothness of the ChIP-seq profile data. Smoothness guaranties that subsequent notes are close by forming a more melodious line. Once randomized it results in random notes, which may be far apart and are perceived by most as not so appealing. Thus, humans can distinguish between smooth signals and signals that change abruptly and perceive the former more appealing than the latter. But clearly there is information in the music that can be exploited by the human ear.

The analyses about differential gene expression and gene clustering use the music as a feature in computer-aided classification tasks. These analyses show that there is some information in the music that a computer can recognize and use for classification. However, a base line using just the ChIP-seq profiles without turning them to music is missing. Without such a base line it remains hard to judge whether the reported results are meaningful or not. Moreover, I think that the classification results shown in Figure 3 are likely to reflect gene expression differences rather than gene function clustering. 

Are the p-values reported in Table 1 for a one- or two-sided test? I do not understand why cluster 2 is not different from all the others except cluster 1.

I think the real potential in chromatin music is not so much in its use in machine learning approaches – it is much better suited for humans learning chromatin states and the like. Humans may be able to recognize patterns that a machine cannot. It would be really interesting to compare a human made chromatin segmentations using chromatin music with a segmentation generated from the ChIP-seq signals by a computer.

Finally, the proposed tool can be used to reach out to the public and demonstrate ideas about epigenomic states etc. using an easily accessible medium such as music.

I have read this submission. I believe that I have an appropriate level of expertise to confirm that it is of an acceptable scientific standard, however I have significant reservations, as outlined above.

# Peer review: Referee response for version 1

**DOI:** 10.5256/f1000research.8610.r12733

**Published:** 2016-03-15

**Authors:** Tao Liu

**Affiliations:** 1 Department of Biochemistry, University at Buffalo, Buffalo, NY, USA; 2 Department of Biostatistics, University at Buffalo, Buffalo, NY, USA

To translate functional genomics data in ChIP-seq to music pieces is entertaining. Cittaro *et al*'s work showed that, the chromatin data which has wider dynamic range is suitable (in my opinion perhaps more suitable than DNA/protein sequences in spite of lacking supporting evidence) for making appealing music. I feel excited while reading the manuscript and listening to the music pieces made by the authors at the same time.This work definitely has the novelty and importance especially for education and popular science. I believe more students and general public will be attracted by the chromatin songs then start to learn the science behind them. Because I don't have the expertise of music theory, I won't comment on the quality of the chromatin music or the methods to tune the music. Since authors showed the music patterns may also reflect the biology on the chromatin and may be associated with gene expression. I will focus on these. 

1. Authors translated chromatin marks ChIP-Seq data from human K562 and NHEK cell lines at refSeq gene bodies from upstream 1k of TSS to 2kb downstream of TES, then tried to study if the music patterns can match the differential gene expression inferred from RNA-Seq.  The prediction power was quite low, as pointed out by the authors, with an AUC of 0.52 ( just a little better than random) with the optimal combination of parameters got from a subset of genes. A natural question is that how well the prediction is by directly using chromatin signals, such as the tags pileup. By comparing with this, we will see whether the underlying biology has been kept or lost during the approach. 
2. Authors showed that the similarities, in terms of hierarchical clustering, of musical representation at gene bodies, from the K562 cell line, can be linked to gene functions and similar gene expression. However I found it's hard to see the consistency of the similarities of music with the similarities of gene function annotations. For example, the cluster 7 and 8 are the closet pair of clusters, although authors claimed that clusters 6,7,8 were linked to regulation of cell cycle, I can't see such words from the word clouds of the middle panel of figure 3.  Instead, I can see that the cluster 1, 3, 5 and 7 all have the same term of biological function 'protein' although they are apart according to the hierarchical clustering. The gene expression analysis is also confusing while looking at the right panel of figure 3 and table 1. It seems that for all the clusters there are two major distributions of gene expression levels from the violin plots -- a big one at log(RPKM) less than 1 and a small one around log(RPKM) of 2. Does that mean the big population of each cluster are just random noises of weakly expressed genes (logRPKM<1)? The numbers of genes in each cluster were also missing in the manuscript. I wonder perhaps the cluster 2 contains very few genes  since although visually it seems the distribution of gene expression of cluster 2 is quite different with all the other clusters, the p-values of the row 2 of table 1 (cluster 2 against 3, 4, 5, 6,7 and 8) are all very small, indicating no significant difference. Additionally, I should bring my comment of point 1 here as well. How the clustering works while checking only the raw ChIP-Seq pileup?
3. Comments on method section:

- For the equations of 'comparison of WAV track', the notations were not explained clearly.
- Description on how the RPKM was calculated is missing. 
- The bases of logarithm functions are missing for the 'log(RPKM)' of gene expression level and the 'absolute logarithm fold change' used to define differential expressed genes.

I have read this submission. I believe that I have an appropriate level of expertise to confirm that it is of an acceptable scientific standard, however I have significant reservations, as outlined above.
